# Income Disparities in Survival and Receipt of Neoadjuvant Chemotherapy and Pelvic Lymph Node Dissection for Muscle-Invasive Bladder Cancer

**DOI:** 10.3390/curroncol31050192

**Published:** 2024-05-02

**Authors:** Ryan M. Antar, Vincent E. Xu, Oluwafolajimi Adesanya, Arthur Drouaud, Noah Longton, Olivia Gordon, Kirolos Youssef, Jad Kfouri, Sarah Azari, Sean Tafuri, Briana Goddard, Michael J. Whalen

**Affiliations:** 1 Department of Urology, School of Medicine, George Washington University, Washington, DC 20052, USA; xuve@gwmail.gwu.edu (V.E.X.); arthurdrouaud@gwmail.gwu.edu (A.D.); kirolos1@gwmail.gwu.edu (K.Y.); jad.kfouri@gwu.edu (J.K.); sazari1992@gmail.com (S.A.); sean.tafuri@gmail.com (S.T.); brianalgoddard@gmail.com (B.G.); mwhalen@mfa.gwu.edu (M.J.W.); 2 School of Medicine, University of Illinois, Urbana-Champaign, Urbana, IL 60612, USA; folajimiadesanya@gmail.com; 3 College of Medicine, Drexel University, Philadelphia, PA 19104, USA; noahlongton@gmail.com

**Keywords:** bladder cancer, MIBC, income, socioeconomic disparities

## Abstract

**Background:** Muscle-invasive bladder cancer (MIBC) is a potentially fatal disease, especially in the setting of locally advanced or node-positive disease. Adverse outcomes have also primarily been associated with low-income status, as has been reported in other cancers. While the adoption of neoadjuvant cisplatin-based chemotherapy (NAC) followed by radical cystectomy (RC) and pelvic lymph node dissection (PLND) has improved outcomes, these standard-of-care treatments may be underutilized in lower-income patients. We sought to investigate the economic disparities in NAC and PLND receipt and survival outcomes in MIBC. **Methods:** Utilizing the National Cancer Database, a retrospective cohort analysis of cT2-4N0-3M0 BCa patients with urothelial histology who underwent RC was conducted. The impact of income level on overall survival (OS) and the likelihood of receiving NAC and PLND was evaluated. **Results:** A total of 25,823 patients were included. This study found that lower-income patients were less likely to receive NAC and adequate PLND (≥15 LNs). Moreover, lower-income patients exhibited worse OS (Median OS 55.9 months vs. 68.2 months, *p* < 0.001). Our findings also demonstrated that higher income, treatment at academic facilities, and recent years of diagnosis were associated with an increased likelihood of receiving standard-of-care modalities and improved survival. **Conclusions:** Even after controlling for clinicodemographic variables, income independently influenced the receipt of standard MIBC treatments and survival. Our findings identify an opportunity to improve the quality of care for lower-income MIBC patients through concerted efforts to regionalize multi-modal urologic oncology care.

## 1. Introduction

Muscle invasive bladder cancer (MIBC) remains a challenge in oncological care, representing approximately 20% of newly diagnosed bladder cancer. It is considered highly aggressive, with the potential for early, distant metastasis, and a poor prognosis. Contemporary studies of multi-modal therapy have demonstrated benefits on overall survival for cisplatin-based neoadjuvant chemotherapy (NAC) in eligible patients, which has led to the adoption of NAC followed by radical cystectomy (RC) and pelvic lymph node dissection (PLND) as the standard of care [1,2]. PLND has become integral to surgical treatment, improving overall survival and disease-specific survival in MIBC, even without frank node-positive disease [3]. Notably, the optimal extent/yield of lymph node dissection remains elusive. However, recent investigations have suggested an optimal lymph node yield of 15 as a potential threshold for improving survival outcomes [4,5,6].

The influence of socioeconomic status (SES) on clinical outcomes in urologic care has also been a critical focus of contemporary research, highlighting notable disparities [7]. SES may influence various aspects of compliance with care, especially given the multi-modal and multi-disciplinary delivery of treatment regimens in MIBC. These include, but are not limited to, shared decision-making, treatment delays, hospital type/resources, insurance accessibility, and financial toxicity [8]. Even after adjusting for such aforementioned social determinants and clinical variables, Washington et al. showed that higher income was associated with an increased likelihood of receiving guideline-based, appropriate MIBC treatment [9]. However, no survival analysis was conducted. Furthermore, a study of 4066 RC patients found that higher income was an independent predictor of receiving complex urinary diversions, even after adjusting for hospital volume and teaching status [10]. Thus, these findings prompt further inquiry into the underlying factors contributing to such income disparities. Indeed, in clinical scenarios without imperative contraindications, all patients diagnosed with MIBC and eligible for treatment should receive a uniform standard of care based on recommended and current guidelines. In this study, we sought to investigate the economic disparities in NAC and PLND receipt and survival outcomes in MIBC in a large, representative national database.

## 2. Materials and Methods

### 2.1. Study Population

This study was conducted by querying the National Cancer Database (NCDB), a hospital-based cancer registry by the Commission on Cancer (CoC) of the American College of Surgeons and the American Cancer Society. This database represents over 1500 Commission-accredited cancer programs in the United States, collecting de-identified data on approximately 70% of all newly diagnosed cancer cases.

A retrospective cohort analysis of patients with cT2-4N0-3M0 urothelial BCa who underwent RC (International Classification of Disease-O-3 (ICD-O-3) organ site codes C67.0-9) with curative intent (e.g., not palliative surgery) between 2004 and 2019 was conducted. Patients were included based on the availability of covariables: Age, Sex, Race, Insurance, Income, Charlson Comorbidity Index (CCI), Facility Type, and Year of Diagnosis. Income status was determined by the median household income for each patient’s zip code of residence and dichotomized as either greater or less than the national median household income. Median household income cutoffs for patients diagnosed from 2000 to 2008, 2008 to 2012, and 2012 to 2016 were USD 35,000, USD 48,000, and USD 50,353, respectively. Income status for patients diagnosed from 2000 to 2008 and 2008 to 2016 was obtained from US Census Data and American Community Survey data, respectively. 

**Figure 1:**
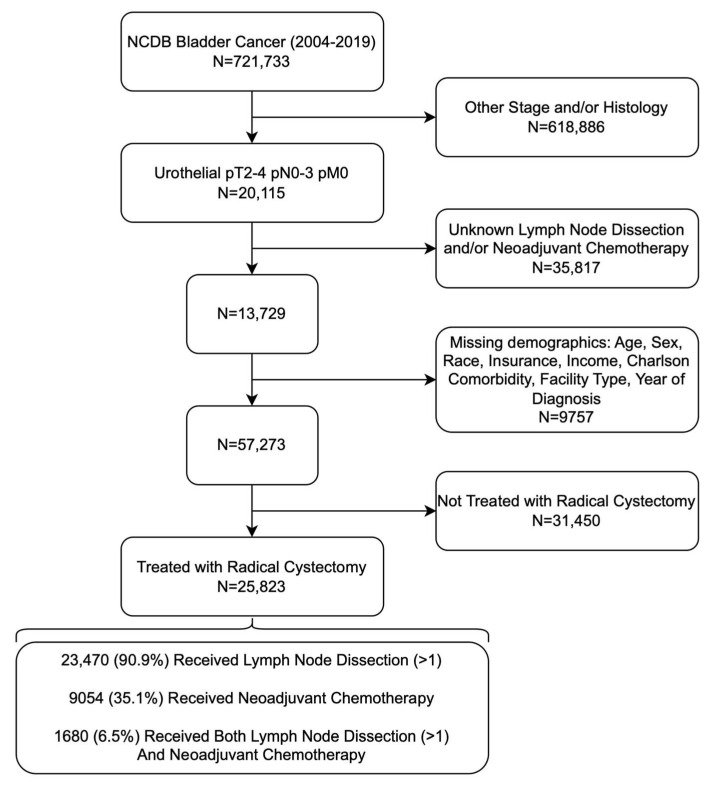
Flow Chart of Inclusion and Exclusion Criteria Used to Isolate Patients with Muscle-Invasive Urothelial Bladder Cancer.

The primary outcomes assessed included receipt of pelvic lymph node dissection and/or neoadjuvant chemotherapy (NAC), as well as Overall Survival (OS). OS was defined as the time from diagnosis to the date of the last follow-up or death. PLND was defined as a regional lymph node yield of ≥1 lymph node (vs. 0 lymph nodes). Adequate PLND was classified as ≥15 regional lymph node yield (vs. <14 lymph nodes). Organizing nodal yield as greater or less than 15 regional nodes examined has been used frequently in prior cystectomy studies [11,12,13]. Of note, only multiagent NAC was considered in our analysis. The inclusion and exclusion criteria used to query our patient population can be visualized in Figure 1.

### 2.2. Statistical Analysis

Descriptive statistics (two-sided independent sample T-tests and Chi-squared tests) were used to compare baseline patient characteristics based on treatment receipt: patients who received RC with NAC and PLND ≥1 and those who did not. 

Univariate and multivariate logistic regressions assessed the likelihood of receipt of NAC, any PLND (≥1), and adequate PLND (≥15). Kaplan–Meier method and log-rank test compared median OS between patients based on income status. Multivariate Cox Proportional Hazards (CPH) regression model adjusted for confounding variables that may relate to overall survival, including age, sex, race/ethnicity, insurance, income, facility type, year of diagnosis, cT stage, cN stage, NAC receipt, and PLND yield. 

All statistical calculations were performed using SPSS software (version 29.0; SPSS Inc., Chicago, IL, USA). All reported *p*-values were based on two-sided hypotheses, with a *p*-value of <0.05 considered statistically significant.

## 3. Results

### 3.1. Patient Baseline Characteristics

Of 25,823 patients, 17,426 underwent RC alone, and 8397 received RC combined with NAC and PLND. The median age was significantly lower in the RC + NAC + PLND group (66 years) compared to the RC group (70 years) (*p*-value < 0.001). There were no statistically significant differences in race and sex between the two treatment groups. A significantly higher proportion of patients had a CCI of zero in the RC + NAC + PLND group (72.3% vs. 66.5% in the RC group, *p*-value < 0.001). A higher percentage of RC + NAC + PLND patients were privately insured (38.5% vs. 29.5% of those receiving RC alone with private insurance) (*p* < 0.001). The income distribution revealed that a larger percentage of patients in the RC + NAC + PLND group fell into the high-income category (64.3%) compared to the RC group (59.1%) (*p* < 0.001). A higher percentage of patients diagnosed between 2012 and 2019 received RC + NAC + PLND than those diagnosed between 2004 and 2011 (81.6% vs. 58.4%, *p*-value < 0.001). These baseline clinicodemographic characteristics are summarized in Table 1. Additionally, clinicodemographic characteristics were further categorized by income status in Table 2.

### 3.2. Likelihood of Receipt of Neoadjuvant Chemotherapy

Several factors influenced the likelihood of receiving NAC in RC patients. Age (continuous) was a significant predictor, with older patients less likely to receive NAC (aOR = 0.961, *p* < 0.001). Sex did not significantly influence the likelihood of receiving NAC. Black patients were less likely to receive NAC compared to white patients (aOR = 0.874, *p* = 0.027). Higher CCI scores were associated with a decreased likelihood of receiving NAC. Uninsured patients were less likely to receive NAC (aOR = 0.737, *p* < 0.001), similar to patients with low income (aOR = 0.803, *p* < 0.001). Low-income status decreased the likelihood of NAC receipt (aOR = 0.803, *p* < 0.001). Patients treated in academic facilities were more likely to receive NAC (aOR = 1.262, *p* < 0.001). The likelihood of receiving NAC significantly increased in recent years (2012–2019, aOR = 3.273, *p* < 0.001). A summary of these results is provided in Table 3.

### 3.3. Likelihood of Any or Adequate Pelvic Lymph Node Dissection

Older patients were less likely to receive PLND of ≥1 and ≥15 lymph nodes (aOR = 0.984 and 0.982, *p* < 0.001). Patient sex and race did not significantly influence the likelihood of PLND of ≥1 and ≥15 lymph nodes. Patients with CCI of one were more likely to receive any PLND (aOR = 1.151, *p* = 0.011) but less likely to receive PLND ≥15 lymph nodes (aOR = 0.892, *p* < 0.001). Low-income status decreased the likelihood of PLND ≥1 lymph node (aOR = 0.905, *p* = 0.027) and PLND ≥ 15 lymph nodes (aOR = 0.903, *p* < 0.001). Patients were more likely to receive PLND ≥ 1 and ≥15 lymph nodes if diagnosed in recent years (2012–2019 vs. 2004–2011) or treated at an academic facility. cT4 tumors decreased the likelihood of PLND ≥1 (aOR = 0.653, *p* < 0.001) and ≥15 (aOR = 0.861, *p* = 0.006). cN1+ status increased the likelihood of PLND ≥1 (aOR = 1.768, *p* < 0.001) but not PLND ≥ 15. Our analysis of logistic regressions is presented in Table 4.

### 3.4. Survival Analysis

**Figure 2:**
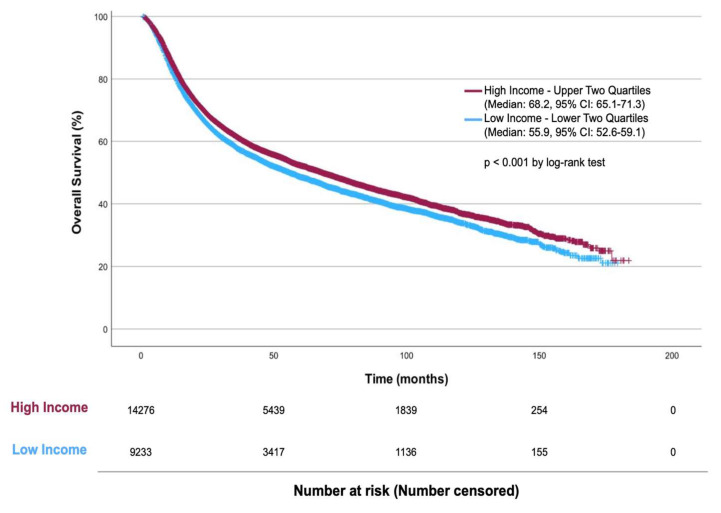
Kaplan–Meier Plot of Overall Survival by Income in MIBC Patients Treated with RC.

The median follow-up time was 35.9 months. Patients with higher income status had greater OS than patients with lower income status (Median OS 68.2 months vs. 55.9 months, *p* < 0.001) (Figure 2). In multivariate CPH, lower income was associated with worse survival (HR = 1.088, *p* < 0.001) (Table 5). Older age (continuous) was associated with worse survival (HR = 1.026, *p* < 0.001). Sex did not significantly impact survival (*p* = 0.315). Higher CCI scores were associated with worse survival. Insurance status showed that patients with private insurance had slightly better survival than federal insurance (HR = 0.931, *p* = 0.003). Patients treated in academic facilities had better overall survival than those in non-academic facilities (HR = 0.936, *p* < 0.001). Higher cT and cN stages were linked with poorer survival. Receipt of NAC and the extent of PLND were associated with better survival outcomes (HR = 0.835 and HR = 0.757 for ≥15 LNs, both *p* < 0.001).

## 4. Discussion

### 4.1. Summary of Key Findings

Low-income status contributes to a worse prognosis for MIBC patients, similar to other cancer types [14]. While adopting NAC followed by RC and PLND has improved outcomes, this study illuminates the critical nuances surrounding disparities in the treatment landscape and survival outcomes for MIBC patients. Our study is the first to focus on income as a potential driver of this disparity, revealing how income impacts the quality of care, as low-income patients are less likely to receive NAC and PLND with RC. We additionally investigated how income affects the adequacy of PLND (≥15 LN yield), which has not been assessed in prior studies on SES in MIBC. Furthermore, lower-income patients had worse OS in our survival analysis, a disparity persisting even after adjusting for various clinicodemographic factors.

### 4.2. Temporal Trends

Despite the 5–10% absolute survival benefit related to receipt of NAC + RC + PLND in MIBC patients [15], its utilization has been historically low, ranging from 15.3 to 34% [16,17,18], depending on the cohort examined. However, the temporal trend of utilization demonstrates an increase from 11 to 24.8% between 2004 and 2011 and from 22.9 to 32.3% between 2011 and 2015 [18,19]. In line with these other findings, our analysis revealed an NAC + RC + PLND utilization rate of 32.5% over the study period, with over a 3-fold increased utilization amongst patients diagnosed from 2012 to 2019. This rise in utilization likely correlates with ongoing updates in treatment guidelines, potentially contributing to our observed improvement in OS in recent years [20,21].

It is crucial to recognize that the goal for NAC + RN + PLND usage should not necessarily be 100%, as the shared decision to utilize these treatments depends on various factors. These include patient comorbidities, tumor characteristics, personal preferences, and shared decision-making with the treating provider. Considering these variables, Vemana et al. estimated that only 42–71% of RC patients would be eligible for neoadjuvant chemotherapy [22]. This may certainly be impacted by chemoradiation as well. Even with a conservative estimate and the upward trend notwithstanding, NAC and PLND remain underutilized in MIBC management, pointing to a conspicuous gap between clinical guidelines and actual practice.

### 4.3. Factors Influencing the Use of NAC and PLND in RC

Disparities in the management of MIBC across socioeconomic lines have been well-documented. Previous reports indicate that older age, non-Hispanic Black race, lower socioeconomic status, and uninsured or Medicaid insurance status have been significantly associated with reduced receipt of definitive care in bladder cancer patients [23,24,25]. In line with these reports, our analyses identified older age, higher Charlson comorbidity scores, insurance status, facility type, and year of diagnosis as significantly impacting overall survival in MIBC. Notably, neither race nor gender was found to be significant in our series. NAC + RC + PLND is the standard of care for MIBC [21], with numerous investigations demonstrating its superiority over radical cystectomy alone in promoting complete excision of occult metastasis at the time of surgery and preventing recurrence [26,27,28]. 

Similarly, the performance and extent of PLND have been directly associated with improved MIBC survival [29,30,31]. Nodal metastasis in MIBC, occurring in approximately 25–30% of cases, is a significant adverse prognostic factor [32,33,34]. Thus, this underscores the critical role of PLND in MIBC treatment to reduce the risk of undiagnosed and, therefore, untreated lymphatic metastasis [35]. The most recent American Urological Association and National Comprehensive Cancer Network guidelines for MIBC emphasize bilateral PLND of the external and internal iliac and obturator lymph nodes [21,36]. While the value of PLND (≥1 lymph node) in improving oncologic outcomes is well established [37,38,39,40], the specific benefits of adequate PLND templates and increased PLND yield remain subjects of debate. Although studies suggest adequate PLND templates enhance survival [41,42,43], the NCDB lacks detailed anatomical PLND template data. Nonetheless, a higher lymph node yield, potentially serving as a proxy for PLND extent, is consistently linked with improved survival. We adopted a threshold of 15 lymph nodes for extensive PLND based on previously reported optimal survival outcomes [4,5,6]. However, it is worth mentioning that this is still under active investigation and based on recent findings from the SWOG S1011 trial, extensive PLND may not provide improved survival compared to standard PLND.

Despite the established importance of PLND in RC for MIBC, disparities in its implementation persist. Understanding the nuanced factors influencing PLND in those undergoing radical cystectomy is vital for optimizing treatment decisions and enhancing outcomes in MIBC management. Our study builds upon previous findings, such as those by Durson et al., who utilized the NCDB to report a reduced likelihood of adequate PLND in patients from urban–rural remote areas [44]. We found that a lower income significantly decreased the probability of receiving any or extensive PLND. Interestingly, while insurance status did not notably affect the receipt of any PLND (≥1 LN), disparities in receiving extensive PLND (≥15 LNs) were evident based on insurance. Furthermore, hospital setting was important, with academic centers over twice as likely to perform both PLND and extensive PLND. These discrepancies suggest that regionalization of care significantly influences the receipt of standard PLND. In contrast, more extensive dissection may be influenced by insurance type, which certainly carries the potential for significant concern.

Our analysis identified multiple factors associated with the underutilization of NAC, including older age, non-Hispanic Black race, a higher CCI, uninsured status, lower income, and treatment in non-academic facilities. These observations align with prior investigations, which reported lower NAC utilization rates among cystectomy patients with similar characteristics—notably lower income [20]. A National Inpatient Sample study by Hoen et al. found that NAC was associated with shorter length of stay but higher total hospital costs [45]. The cost of NAC administration, in addition to radical cystectomy, estimated at a mean of USD 52,429 by Stevenson et al. [46], may present a considerable financial barrier for low-income groups, with potentially adverse consequences on survival. Given that lower-income patients had a 19.7% lower chance of receiving NAC in our study, it is crucial to recognize and mitigate this financial toxicity. A 2019 cost analysis evaluated 2693 patients undergoing MIBC treatments, comprising nearly 88% White patients, with Black and Hispanic patients representing only 4.4% and 3.3%, respectively [47]. This racial distribution in treatment modalities could reflect the baseline demographics of those diagnosed with MIBC. These proportions highlight concerning disparities and accentuate the broader racial wealth gap experienced by Black individuals, particularly those with lower incomes and less extensive insurance coverage [48]. Moreover, to explore precedent in another disease state, Oake et al. found that after adjusting for demographic and clinicopathological factors, the top income quintiles were 2.3 times more likely than the bottom income quintile to choose and receive radical prostatectomy surgery as opposed to radical radiotherapy for prostate cancer treatment [49]. Our study found lower-income patients to have reduced administration of NAC + RC + PLND treatments and worsened overall survival by over 12 months. These findings illuminate the intersectionality of social determinants of health, such as income, on treatment decision-making. It is crucial for truly informed shared decision-making to incorporate discussion of these elements. Further research is necessary to define the optimal approach/questionnaires in this domain. 

### 4.4. Policy Implications and Recommendations for Practice

Our analysis and prior studies have shown that socioeconomic disparities’ influence on access to care and OS estimates among MIBC patients is considerable. There is an urgent need to address these systemic issues to improve access to life-saving care for all patients regardless of income status, to lower the morbidity and mortality burden associated with MIBC, and to ensure equity in healthcare delivery. Considering the higher additional costs of NAC + RC, compared to RC alone [46], it can be argued that the cost of care is a significant source of disparities in access and OS among MIBC patients. Unsurprisingly, uninsured patients were over 30% less likely to receive NAC. Among insured patients, those with private insurance exhibited marginally higher OS estimates than those insured by Medicare and Medicaid. These results indicate that by lowering the cost of care and increasing access to standard of care for MIBC patients of lower income status, OS from MIBC may be dramatically improved. 

This may be achieved by policy initiatives focused on expanding affordable access to health insurance for patients of lower socioeconomic status while expanding coverage of publicly funded insurance programs to include NAC + RC to reduce out-of-pocket payments for those most in need. Previous evidence supports this as a viable strategy. Michel et al. showed that expanding Medicaid insurance coverage was associated with a simultaneous decrease in the uninsured rate, late-stage diagnosis of genitourinary cancers, and an increase in the proportion of patients receiving active surveillance for low-risk prostate cancer [50]. Interestingly, their analyses found this association stronger among patients of lower income status, pointing to its viability as a targeted approach to reducing socioeconomic disparities in care access. Further evidence of the impact of increased insurance coverage on alleviating disparities in MIBC care access was provided by Jiang et al., who showed that expansion of Medicaid coverage by the Affordable Care Act (ACA) significantly reduced racial disparities in timely care access to MIBC care by 13.7% [51]. 

In addition, our analysis found that patients treated at academic facilities were more likely to receive NAC, any PLND, adequate PLND, and improved OS. These findings are reminiscent of previously reported benefits of academic hospitals, including enhanced patient access to cutting-edge treatments and specialized standards of care, which may not be readily available in private practice settings [52]. Similarly, in academic medical centers, surgeons are more likely to work within multidisciplinary teams involving different sub-specialists, offering a collaborative approach to patient care that may be lacking in a private practice setting [53,54]. This may promote the provision of standard of care, including NAC, to MIBC patients treated in academic medical facilities. By encouraging increased uptake of multidisciplinary and collaborative working environments among private practice providers, the gap in standard-of-care access noted in our analysis could be bridged, with consequent benefits for OS. 

In recent years, the relationship between distance traveled to obtain care and care outcomes has been investigated, resulting in the proposition of regionalization of cancer care as a viable strategy to improve outcomes in cancer patients. This concept originates from studies reporting lower postoperative morbidity and mortality among patients treated in high-volume facilities [55,56]. This has resulted in the push to develop regional cancer care centers, which are more likely to be academic facilities. This approach, however, has its limitations, the most important of which would be the increased distance between patients’ homes and the treatment facility. An increased distance to access care has been associated with a decrease in utilization of cancer care [57,58] and an increased chance of metastatic disease at cancer diagnosis [59,60]. Given these contradictory findings, Ryan et al. conducted a retrospective cohort study investigating the relationship between mortality and distance to treatment facility in MIBC patients [61]. The authors report an inverse relationship between distance to the treatment center from patient residence and overall mortality, a significant result especially for those treated at academic centers, thus supporting the case for regionalizing MIBC care. Moreover, Maurice et al. found that treatment at academic institutions influenced urinary diversion utilization in radical cystectomy [10]. Further studies may be required to clarify contradictions and strengthen the evidence behind this concept.

Education level is a widely used alternative metric for assessing socioeconomic status, with educational attainment correlating inversely with socioeconomic status [62]. While we have not explicitly reported this, several studies have demonstrated a higher incidence of MIBC in patients with lower educational attainment [9,63,64]. This provides a unique opportunity for patient education programs focused on low-income and less-educated populations to improve their knowledge of the symptoms, risk factors, and best treatment options. This would effectively empower such patients to seek and/or demand the highest level of care, bridging gaps in care access and ultimately improving overall survival estimates. This approach is practical in general oncological care by facilitating earlier symptom recognition, cancer diagnosis, treatment adherence, and improved outcomes [65].

By implementing ongoing surveys designed to track utilization of MIBC standard of care among socioeconomically disadvantaged patients, policymakers and practice providers can identify areas requiring intervention and deploy healthcare resources efficiently to help improve equity in MIBC care access and increase overall survival for all.

### 4.5. Study Limitations

Our study is not without limitations, the first being the retrospective nature of our analysis and inherent limitations of the NCDB, which may be prone to selection and misclassification bias. We have avoided these by excluding patients with insufficient data points from our final patient cohort. Given limitations within the NCDB, we were only able to assess for overall survival and not cancer-specific survival, among other survival analyses. Additionally, the NCDB certainly does not capture patient and surgeon preferences in the decision to undergo various treatment modalities. Further, our use of income as the sole metric for socioeconomic status may be interpreted as insufficient as socioeconomic status is influenced by more than patient income figures. However, we controlled for confounding variables within the NCDB and aimed to investigate the independent influence of income on access to MIBC standard of care and overall survival in our patient cohort specifically. Existing data show income as an independent predictor of the choice of intervention among urological patients and, further, even influencing the choice of urinary diversion in RC patients [10]. 

## 5. Conclusions

Neoadjuvant chemotherapy and pelvic lymphadenectomy are underutilized in lower-income muscle-invasive bladder cancer patients, beyond expectations of medical ineligibility for neoadjuvant chemotherapy. Without these standard-of-care modalities, our study demonstrated that lower-income patients have worse overall survival. Our findings identify an opportunity to improve the quality of care for lower-income MIBC patients through concerted efforts to regionalize multi-modal urologic oncology care.

## Figures and Tables

**Table 1:** Baseline Characteristics of Patients with Urothelial Bladder Cancer Treated with Radical Cystectomy with or without Neoadjuvant Chemotherapy and Pelvic Lymph Node Dissection.

	Treatment Receipt		
	RC	RC + NAC + PLND	*p*-Value *
	Count (%)	Count (%)	
Median Age	70	66	**<0.001**
Sex			0.864
Male	13,226, 75.9%	6365, 75.8%	
Female	4200, 24.1%	2032, 24.2%	
Race			0.909
White	15,563, 89.3%	7513, 89.5%	
Black	993, 5.7%	468, 5.6%	
Other	870, 5.0%	416, 5.0%	
CCI			**<0.001**
0	11,597, 66.5%	6069, 72.3%	
1	3933, 22.6%	1564, 18.6%	
2	1304, 7.5%	496, 5.9%	
3+	592, 3.4%	268, 3.2%	
Insurance			**<0.001**
No Insurance	391, 2.2%	185, 2.2%	
Private	5133, 29.5%	3232, 38.5%	
Medicaid	777, 4.5%	470, 5.6%	
Medicare	11,125, 63.8%	4510, 53.7%	
Income			**<0.001**
Low Income	7132, 40.9%	2994, 35.7%	
High Income	10,294, 59.1%	5403, 64.3%	
Facility Type			**<0.001**
Non-Academic	9117, 52.3%	3720, 44.3%	
Academic	8309, 47.7%	4677, 55.7%	
Year of Diagnosis			**<0.001**
2004–2011	7246, 41.6%	1544, 18.4%	
20012–2019	10,180, 58.4%	6853, 81.6%	
cT Stage			**0.009**
2	14,187, 81.4%	6702, 79.8%	
3	2011, 11.5%	1045, 12.4%	
4	1228, 7.0%	650, 7.7%	
cN Stage			**<0.001**
0	16,336, 93.7%	7532, 89.7%	
1	547, 3.1%	457, 5.4%	
2	463, 2.7%	324, 3.9%	
3	80, 00.5%	84, 1.0%	

* Significance was calculated with a 2-sided independent sample *t*-test and Chi-square. Abbreviations used: Radical Cystectomy (RC); Neoadjuvant Chemotherapy (NAC); Pelvic Lymph Node Dissection (PLND); Charlson Comorbidity Index (CCI). Values of *p* < 0.05 bolded for statistical significance.

**Table 2:** Baseline Characteristics of Patients with Urothelial Bladder Cancer Treated with Radical Cystectomy by Income Status.

	Income Status Split by Median Income		
	High Income	Low Income	*p*-Value *
	Count (%)	Count (%)	
Median Age	69	68	**<0.001**
Sex			0.572
Male	11,928, 76.0%	7663, 75.7%	
Female	3769, 24.0%	2463, 24.3%	
Race			**<0.001**
White	14,397, 91.7%	8679, 85.7%	
Black	495, 3.2%	966, 9.5%	
Other	805, 5.1%	481, 4.8%	
CCI			**<0.001**
0	10,938, 69.7%	6728, 66.4%	
1	3185, 20.3%	2312, 22.8%	
2	1062, 6.8%	738, 7.3%	
3+	512, 3.3%	348, 3.4%	
Insurance			**<0.001**
No Insurance	257, 1.6%	319, 3.2%	
Private	5479, 34.9%	2886, 28.5%	
Medicaid	578, 3.7%	669, 6.6%	
Medicare	9383, 59.8%	6252, 61.7%	
Facility Type			**<0.001**
Non-Academic	7671, 48.9%	5166, 51.0%	
Academic	8026, 51.1%	4960, 49.0%	
Year of Diagnosis			**<0.001**
2004–2011	5220, 33.3%	3570, 35.3%	
20012–2019	10,477, 66.7%	6556, 64.7%	
cT Stage			0.31
2	12,743, 81.2%	8146, 80.4%	
3	1837, 11.7%	1219, 12.0%	
4	1117, 7.1%	761, 7.5%	
cN Stage			0.772
0	14,516, 92.5%	9352, 92.4%	
1	612, 3.9%	392, 3.9%	
2	466, 3.0%	321, 3.2%	
3	103, 0.7%	61, 0.6%	
Chemotherapy			**<0.001**
No NAC	9900, 63.1%	6869, 67.8%	
NAC	5797, 36.9%	3257, 32.2%	
PLND			**<0.001**
0 LNs	1371, 8.7%	982, 9.7%	
1–14 LNs	6989, 44.5%	4739, 47.8%	
15+ LNs	7337, 46.7%	4405, 43.5%	

* Significance was calculated with a 2-sided independent sample *t*-test and Chi-square. Abbreviations used: Radical Cystectomy (RC); Neoadjuvant Chemotherapy (NAC); Pelvic Lymph Node Dissection (PLND); Charlson Comorbidity Index (CCI). Values of *p* < 0.05 bolded for statistical significance.

**Table 3:** Multivariate Logistic Regression Predicting the Likelihood of Receipt of Neoadjuvant Chemotherapy.

	Univariate		Multivariate	
	OR (CI)	*p*-Value	aOR (CI)	*p*-Value
Age (continuous)	0.963 (0.961–0.966)	**<0.001**	0.961 (0.957–0.964)	**<0.001**
Sex (Ref = Male)				
Female	0.990 (0.932–1.051)	0.736	1.009 (0.947–1.075)	0.786
Race (Ref = White)				
Black	0.984 (0.881–1.100)	0.782	0.874 (0.777–0.985)	**0.027**
Other	0.961 (0.853–1.081)	0.504	0.820 (0.724–0.928)	**0.002**
CCI (Ref = 0)				
1	0.743 (0.696–0.793)	**<0.001**	0.838 (0.782–0.897)	**<0.001**
2	0.698 (0.628–0.776)	**<0.001**	0.744 (0.666–0.831)	**<0.001**
3	0.838 (0.725–0.969)	**0.017**	0.820 (0.705–0.954)	**0.01**
Insurance (Ref = Federal)				
Private	1.501 (1.422–1.585)	**<0.001**	0.989 (0.924–1.058)	0.989
Uninsured	1.106 (0.928–1.318)	0.259	0.737 (0.588–0.860)	**<0.001**
Income (Ref = High Income)				
Low Income	0.810 (0.768–0.854)	**<0.001**	0.803 (0.759–0.850)	**<0.001**
Facility Type (Ref = Non-Academic)				
Academic	1.295 (1.230–1.363)	**<0.001**	1.262 (1.196–1.332)	**<0.001**
Year of Diagnosis (Ref = 2004–2011)				
2012–2019	3.061 (2.881–3.252)	**<0.001**	3.273 (3.073–3.485)	**<0.001**
cT (Ref = 2)				
3	1.083 (1.001–1.172)	**0.047**	1.123 (1.032–1.223)	**0.008**
4	1.209 (1.097–1.332)	**<0.001**	1.215 (1.094–1.349)	**<0.001**
cN (Ref = 0)				
1	1.823 (1.611–2.075)	**<0.001**	1.750 (1.528–2.005)	**<0.001**
2	1.463 (1.267–1.689)	**<0.001**	1.398 (1.198–1.631)	**<0.001**
3	2.351 (1.727–3.202)	**<0.001**	1.702 (1.233–2.349)	**0.001**

Multivariate model summary: Odds Ratios (OR), Adjusted Odds Ratios (aOR), and 95% Confidence Intervals (CI) with reference categories in parentheses. Abbreviation used: Radical Cystectomy (RC); Neoadjuvant Chemotherapy (NAC); Charlson Comorbidity Index (CCI). Values of *p* < 0.05 bolded for statistical significance.

**Table 4:** Multivariate Logistic Regressions Predicting the Likelihood of Receipt of Lymph Node Dissection.

	Receipt of PLND (≥1)			
	Univariate		Multivariate	
	OR (CI)	*p*-Value	aOR (CI)	*p*-Value
Age (continuous)	0.983 (0.979–0.988)	**<0.001**	0.984 (0.978–0.989)	**<0.001**
Sex (Ref = Male)				
Female	1.002 (0.908–1.107)	0.965	1.025 (0.927–1.134)	0.626
Race (Ref = White)				
Black	1.002 (0.834–1.204)	0.981	0.898 (0.743–1.085)	0.264
Other	1.022 (0.840–1.244)	0.828	0.860 (0.704–1.050)	0.138
CCI (Ref = 0)				
1	1.071 (0.963–1.191)	0.208	1.151 (1.033–1.283)	**0.011**
2	1.075 (0.905–1.275)	0.411	1.144 (0.962–1.362)	0.128
3	1.159 (0.903–1.487)	0.247	1.176 (0.913–1.514)	0.21
Insurance (Ref = Federal)				
Private	1.185 (1.079–1.301)	**<0.001**	0.985 (0.881–1.101)	0.784
Uninsured	1.090 (0.814–1.459)	0.564	0.904 (0.665–1.228)	0.518
Income (Ref = High Income)				
Low Income	0.891 (0.818–0.971)	**0.009**	0.905 (0.829–0.989)	**0.027**
Facility Type (Ref = Non-Academic)				
Academic	2.348 (2.145–2.570)	**<0.001**	2.340 (2.135–2.563)	**<0.001**
Year of Diagnosis (Ref = 2004–2011)				
2012–2019	1.546 (1.419–1.685)	**<0.001**	1.538 (1.409–1.679)	**<0.001**
cT (Ref = 2)				
3	0.994 (0.870–1.136)	0.93	0.962 (0.839–1.102)	0.576
4	0.720 (0.621–0.834)	**<0.001**	0.653 (0.560–0.760)	**<0.001**
cN (Ref = 0)				
1	1.693 (1.294–2.215)	**<0.001**	1.768 (1.344–2.325)	**<0.001**
2	2.046 (1.473–2.843)	**<0.001**	2.290 (1.638–3.203)	**<0.001**
3	1.315 (0.730–2.371)	0.362	1.119 (0.660–2.177)	0.552
	**Receipt of PLND (≥15)**			
	**Univariate**		**Multivariate**	
	**OR (CI)**	***p*-Value**	**aOR (CI)**	***p*-Value**
Age (continuous)	0.979 (0.977–0.982)	**<0.001**	0.982 (0.979–0.985)	**<0.001**
Sex (Ref = Male)				
Female	1.004 (0.946–1.066)	0.883	1.022 (0.960–1.087)	0.5
Race (Ref = White)				
Black	1.002 (0.896–1.119)	0.977	0.911 (0.811–1.023)	0.115
Other	1.056 (0.939–1.188)	0.364	0.902 (0.799–1.018)	0.095
CCI (Ref = 0)				
1	0.829 (0.778–0.883)	**<0.001**	0.892 (0.835–0.952)	**<0.001**
2	0.779 (0.704–0.863)	**<0.001**	0.838 (0.754–0.931)	**<0.001**
3	0.834 (0.723–0.962)	**0.013**	0.864 (0.745–1.001)	0.052
Insurance (Ref = Federal)				
Private	1.362 (1.289–1.440)	**<0.001**	1.103 (1.031–1.180)	**0.004**
Uninsured	1.028 (0.864–1.224)	0.752	0.817 (0.679–0.983)	**0.032**
Income (Ref = High Income)				
Low Income	0.885 (0.840–0.933)	**<0.001**	0.903 (0.855–0.954)	**<0.001**
Facility Type (Ref = Non-Academic)				
Academic	2.115 (2.008–2.229)	**<0.001**	2.118 (2.009–2.233)	**<0.001**
Year of Diagnosis (Ref = 2004–2011)				
2012–2019	1.573 (1.489–1.662)	**<0.001**	1.606 (1.518–1.700)	**<0.001**
cT (Ref = 2)				
3	0.983 (0.908–1.064)	0.673	1.000 (0.920–1.087)	0.994
4	0.882 (0.797–0.975)	**0.014**	0.861 (0.774–0.957)	**0.006**
cN (Ref = 0)				
1	1.127 (0.989–1.284)	0.073	1.104 (0.963–1.266)	0.155
2	1.108 (0.958–1.282)	0.167	1.147 (0.984–1.337)	0.079
3	1.465 (1.059–2.027)	**0.021**	1.254 (0.898–1.750)	0.184

Multivariate model summary: Odds Ratios (OR), Adjusted Odds Ratios (aOR), and 95% Confidence Intervals (CI) with reference categories in parentheses. Abbreviation used: Radical Cystectomy (RC); Pelvic Lymph Node Dissection (PLND); Charlson Comorbidity Index (CCI). Values of *p* < 0.05 bolded for statistical significance.

**Table 5:** Multivariate Cox Proportional Hazards Model for Overall Survival in Patients with Urothelial Bladder Cancer Treated with Radical Cystectomy.

	HR	95% CI	*p*-Value
Age (continuous)	1.026	1.023–1.028	**<0.001**
Sex (Ref = Male)			
Female	1.022	0.980–1.065	0.315
Race (Ref = White)			
Black	1.073	0.991–1.162	0.084
Other	0.913	0.836–0.997	**0.044**
CCI (Ref = 0)			
1	1.213	1.162–1.265	**<0.001**
2	1.451	1.357–1.550	**<0.001**
3	1.539	1.394–1.700	**<0.001**
Insurance (Ref = Federal)			
Private	0.931	0.889–0.976	**0.003**
Uninsured	0.948	0.831–1.083	0.432
Income (Ref = High Income)			
Low Income	1.088	1.049–1.129	**<0.001**
Facility Type (Ref = Non-Academic)			
Academic	0.936	0.902–0.971	**<0.001**
Year of Diagnosis (Ref = 2004–2011)			
2012–2019	0.859	0.827–0.892	**<0.001**
cT (Ref = 2)			
3	1.318	1.250–1.389	**<0.001**
4	1.668	1.566–1.776	**<0.001**
cN (Ref = 0)			
1	1.302	1.191–1.424	**<0.001**
2	1.804	1.647–1.975	**<0.001**
3	1.577	1.277–1.948	**<0.001**
NAC Receipt (Ref = No NAC)			
NAC Treatment	0.835	0.801–0.871	**<0.001**
PLND (Ref = No PLND)			
>0 & <14 LNs	0.93	0.876–0.987	**0.017**
≥ 15 LNs	0.757	0.711–0.806	**<0.001**

Multivariate model summary with reference categories in parentheses. Abbreviation used: Radical Cystectomy (RC); Neoadjuvant Chemotherapy (NAC); Pelvic Lymph Node Dissection (PLND); Charlson Comorbidity Index (CCI). Values of *p* < 0.05 bolded for statistical significance.

## Data Availability

This study utilized data from the National Cancer Database.

## References

[1] Patel V.G., Oh W.K., Galsky M.D. (2020). Treatment of muscle-invasive and advanced bladder cancer in 2020. CA Cancer J. Clin..

[2] Gore J.L., Litwin M.S., Lai J., Yano E.M., Madison R., Setodji C., Adams J.L., Saigal C.S., the Urologic Diseases in America Project (2010). Use of radical cystectomy for patients with invasive bladder cancer. J. Natl. Cancer Inst..

[3] Morgan T.M., Barocas D.A., Penson D.F., Chang S.S., Ni S., Clark P.E., Smith J.A., Cookson M.S. (2012). Lymph node yield at radical cystectomy predicts mortality in node-negative and not node-positive patients. Urology.

[4] May M., Herrmann E., Bolenz C., Brookman-May S., Tiemann A., Moritz R., Fritsche H.M., Burger M., Trojan L., Michel M.S. (2011). Association between the number of dissected lymph nodes during pelvic lymphadenectomy and cancer-specific survival in patients with lymph node-negative urothelial carcinoma of the bladder undergoing radical cystectomy. Ann. Surg. Oncol..

[5] Li R., Petros F.G., Davis J.W. (2018). Extended Pelvic Lymph Node Dissection in Bladder Cancer. J. Endourol..

[6] Leminski A., Kaczmarek K., Michalski W., Malkiewicz B., Kotfis K., Slojewski M. (2021). The Influence of Lymph Node Count on Oncological Outcome of Radical Cystectomy in Chemotherapy Pre-Treated and Chemotherapy-Naive Patients with Muscle Invasive Bladder Cancer. J. Clin. Med..

[7] Golombos D.M., O’Malley P., Lewicki P., Nguyen D.P., Stone B.V., Al Hussein Al Awamlh B., Scherr D.S. (2017). The impact of socioeconomic status on perioperative complications and oncologic outcomes in patients undergoing radical cystectomy. World J. Urol..

[8] Russell B., Haggstrom C., Holmberg L., Liedberg F., Gardmark T., Bryan R.T., Kumar P., Van Hemelrijck M. (2021). Systematic review of the association between socioeconomic status and bladder cancer survival with hospital type, comorbidities, and treatment delay as mediators. BJUI Compass.

[9] Washington S.L., Neuhaus J., Meng M.V., Porten S.P. (2019). Social Determinants of Appropriate Treatment for Muscle-Invasive Bladder Cancer. Cancer Epidemiol. Biomark. Prev..

[10] Maurice M.J., Kim S.P., Abouassaly R. (2017). Socioeconomic status is associated with urinary diversion utilization after radical cystectomy for bladder cancer. Int. Urol. Nephrol..

[11] Jeong I.G., Park J., Song K., Ro J.Y., Song C., Hong J.H., Ahn H., Kim C.S. (2011). Comparison of 2002 TNM nodal status with lymph node density in node-positive patients after radical cystectomy for bladder cancer: Analysis by the number of lymph nodes removed. Urol. Oncol..

[12] Lerner S. S1011 Standard or Extended Pelvic Lymphadenectomy in Treating Patients Undergoing Surgery for Invasive Bladder Cancer. https://classic.clinicaltrials.gov/ct2/show/NCT01224665..

[13] Herr H., Lee C., Chang S., Lerner S., for the Bladder Cancer Collaborative Group (2004). Standardization of radical cystectomy and pelvic lymph node dissection for bladder cancer: A collaborative group report. J. Urol..

[14] Afshar N., English D.R., Milne R.L. (2021). Factors Explaining Socio-Economic Inequalities in Cancer Survival: A Systematic Review. Cancer Control.

[15] Advanced Bladder Cancer Meta-analysis Collaboration (2005). Neoadjuvant chemotherapy in invasive bladder cancer: Update of a systematic review and meta-analysis of individual patient data advanced bladder cancer (ABC) meta-analysis collaboration. Eur. Urol..

[16] van Hoogstraten L.M.C., Man C.C.O., Witjes J.A., Meijer R.P., Mulder S.F., Smilde T.J., Ripping T.M., Kiemeney L.A., Aben K.K.H., BlaZIB Study Group (2023). Low adherence to recommended use of neoadjuvant chemotherapy for muscle-invasive bladder cancer. World J. Urol..

[17] Grant C.M., Amdur R., Whalen M.J. (2019). Trends in the use of neoadjuvant chemotherapy for bladder cancer with nonurothelial variant histology: An analysis of the National Cancer Database. Indian J. Urol..

[18] Nyame Y.A., Holt S.K., Winters B., Psutka S.P., Dash A., Schade G.R., Lin D.W., Yu E.Y., Gore J.L., Wright J.L. (2019). Neoadjuvant chemotherapy utilization in muscle-invasive bladder cancer: Increasing yet inappropriate use?. J. Clin. Oncol..

[19] McFerrin C., Davaro F., May A., Raza S., Siddiqui S., Hamilton Z. (2020). Trends in utilization of neoadjuvant and adjuvant chemotherapy for muscle invasive bladder cancer. Investig. Clin. Urol..

[20] Duplisea J.J., Mason R.J., Reichard C.A., Li R., Shen Y., Boorjian S.A., Dinney C.P. (2019). Trends and disparities in the use of neoadjuvant chemotherapy for muscle-invasive urothelial carcinoma. Can. Urol. Assoc. J..

[21] Chang S.S., Bochner B.H., Chou R., Dreicer R., Kamat A.M., Lerner S.P., Lotan Y., Meeks J.J., Michalski J.M., Morgan T.M. (2017). Treatment of Non-Metastatic Muscle-Invasive Bladder Cancer: AUA/ASCO/ASTRO/SUO Guideline. J. Urol..

[22] Vemana G., Nepple K.G., Vetter J., Sandhu G., Strope S.A. (2014). Defining the potential of neoadjuvant chemotherapy use as a quality indicator for bladder cancer care. J. Urol..

[23] Shah A.A., Sun Z., Eom K.Y., Grajales V., Pekala K.R., Davies B.J., Jacobs B.L., Sabik L.M. (2022). Treatment disparities in muscle-invasive bladder cancer: Evidence from a large statewide cancer registry. Urol. Oncol..

[24] Knorr J.M., Campbell R.A., Cockrum J., Dalton J.E., Murthy P.B., Berglund R.K., Cullen J., Weight C.J., Almassi N., Abouassaly R. (2022). Neighborhood Socioeconomic Disadvantage Associated with Increased 90-Day Mortality Following Radical Cystectomy. Urology.

[25] Gray P.J., Fedewa S.A., Shipley W.U., Efstathiou J.A., Lin C.C., Zietman A.L., Virgo K.S. (2013). Use of potentially curative therapies for muscle-invasive bladder cancer in the United States: Results from the National Cancer Data Base. Eur. Urol..

[26] Posielski N., Koenig H., Ho O., Porter C., Flores J.P. (2022). Use of Neoadjuvant Chemotherapy in Elderly Patients with Muscle-Invasive Bladder Cancer: A Population-Based Study, 2006–2017. Oncology.

[27] Hamid A., Ridwan F.R., Parikesit D., Widia F., Mochtar C.A., Umbas R. (2020). Meta-analysis of neoadjuvant chemotherapy compared to radical cystectomy alone in improving overall survival of muscle-invasive bladder cancer patients. BMC Urol..

[28] Grossman H.B., Natale R.B., Tangen C.M., Speights V.O., Vogelzang N.J., Trump D.L., deVere White R.W., Sarosdy M.F., Wood D.P., Raghavan D. (2003). Neoadjuvant chemotherapy plus cystectomy compared with cystectomy alone for locally advanced bladder cancer. N. Engl. J. Med..

[29] Honma I., Masumori N., Sato E., Maeda T., Hirobe M., Kitamura H., Takahashi A., Itoh N., Tamakawa M., Tsukamoto T. (2006). Removal of more lymph nodes may provide better outcome, as well as more accurate pathologic findings, in patients with bladder cancer—analysis of role of pelvic lymph node dissection. Urology.

[30] Konety B.R., Joslyn S.A. (2003). Factors influencing aggressive therapy for bladder cancer: An analysis of data from the SEER program. J. Urol..

[31] Malkiewicz B., Kielb P., Gurwin A., Knecht K., Wilk K., Dobruch J., Zdrojowy R. (2021). The Usefulness of Lymphadenectomy in Bladder Cancer-Current Status. Medicina.

[32] Lerner S.P., Skinner D.G., Lieskovsky G., Boyd S.D., Groshen S.L., Ziogas A., Skinner E., Nichols P., Hopwood B. (1993). The rationale for en bloc pelvic lymph node dissection for bladder cancer patients with nodal metastases: Long-term results. J. Urol..

[33] Moschini M., Arbelaez E., Cornelius J., Mattei A., Shariat S.F., Dell Oglio P., Zaffuto E., Salonia A., Montorsi F., Briganti A. (2018). Pattern of node metastases in patients treated with radical cystectomy and extended or superextended pelvic lymph node dissection due to bladder cancer. Urol. Oncol..

[34] Bruins H.M., Skinner E.C., Dorin R.P., Ahmadi H., Djaladat H., Miranda G., Cai J., Daneshmand S. (2014). Incidence and location of lymph node metastases in patients undergoing radical cystectomy for clinical non-muscle invasive bladder cancer: Results from a prospective lymph node mapping study. Urol. Oncol..

[35] Wright J.L., Lin D.W., Porter M.P. (2008). The association between extent of lymphadenectomy and survival among patients with lymph node metastases undergoing radical cystectomy. Cancer.

[36] National Comprehensive Cancer Network Bladder Cancer. https://www.nccn.org/guidelines/guidelines-detail?category=1&id=1417.

[37] Karl A., Carroll P.R., Gschwend J.E., Knuchel R., Montorsi F., Stief C.G., Studer U.E. (2009). The impact of lymphadenectomy and lymph node metastasis on the outcomes of radical cystectomy for bladder cancer. Eur. Urol..

[38] Konety B.R., Joslyn S.A., O’Donnell M.A. (2003). Extent of pelvic lymphadenectomy and its impact on outcome in patients diagnosed with bladder cancer: Analysis of data from the Surveillance, Epidemiology and End Results Program data base. J. Urol..

[39] Stein J.P., Cai J., Groshen S., Skinner D.G. (2003). Risk factors for patients with pelvic lymph node metastases following radical cystectomy with en bloc pelvic lymphadenectomy: Concept of lymph node density. J. Urol..

[40] Abdollah F., Sun M., Schmitges J., Djahangirian O., Tian Z., Jeldres C., Perrotte P., Shariat S.F., Montorsi F., Karakiewicz P.I. (2012). Stage-specific impact of pelvic lymph node dissection on survival in patients with non-metastatic bladder cancer treated with radical cystectomy. BJU Int..

[41] Bi L., Huang H., Fan X., Li K., Xu K., Jiang C., Liu H., Dong W., Zhang S., Yang X. (2014). Extended vs non-extended pelvic lymph node dissection and their influence on recurrence-free survival in patients undergoing radical cystectomy for bladder cancer: A systematic review and meta-analysis of comparative studies. BJU Int..

[42] Leissner J., Ghoneim M.A., Abol-Enein H., Thuroff J.W., Franzaring L., Fisch M., Schulze H., Managadze G., Allhoff E.P., El-Baz M.A. (2004). Extended radical lymphadenectomy in patients with urothelial bladder cancer: Results of a prospective multicenter study. J. Urol..

[43] Dangle P.P., Gong M.C., Bahnson R.R., Pohar K.S. (2010). How do commonly performed lymphadenectomy templates influence bladder cancer nodal stage?. J. Urol..

[44] Dursun F., Elshabrawy A., Wang H., Kaushik D., Liss M.A., Svatek R.S., Gore J.L., Mansour A.M. (2023). Impact of rural residence on the presentation, management and survival of patients with non-metastatic muscle-invasive bladder carcinoma. Investig. Clin. Urol..

[45] Hoeh B., Flammia R.S., Hohenhorst L., Sorce G., Chierigo F., Panunzio A., Tian Z., Saad F., Gallucci M., Briganti A. (2022). Effect of Neoadjuvant Chemotherapy on Complications, in-Hospital Mortality, Length of Stay and Total Hospital Costs in Bladder Cancer Patients Undergoing Radical Cystectomy. Cancers.

[46] Stevenson S.M., Danzig M.R., Ghandour R.A., Deibert C.M., Decastro G.J., Benson M.C., McKiernan J.M. (2014). Cost-effectiveness of neoadjuvant chemotherapy before radical cystectomy for muscle-invasive bladder cancer. Urol. Oncol..

[47] Williams S.B., Shan Y., Ray-Zack M.D., Hudgins H.K., Jazzar U., Tyler D.S., Freedland S.J., Swanson T.A., Baillargeon J.G., Hu J.C. (2019). Comparison of Costs of Radical Cystectomy vs Trimodal Therapy for Patients with Localized Muscle-Invasive Bladder Cancer. JAMA Surg..

[48] Boen C., Keister L., Aronson B. (2020). Beyond Net Worth: Racial Differences in Wealth Portfolios and Black-White Health Inequality across the Life Course. J. Health Soc. Behav..

[49] Oake J.D., Harasemiw O., Tangri N., Ferguson T.W., Saranchuk J.W., Bansal R.K., Drachenberg D.E., Nayak J.G. (2021). The Association between Income Status and Treatment Selection for Prostate Cancer in a Universal Health Care System: A Population-Based Analysis. J. Urol..

[50] Michel K.F., Spaulding A., Jemal A., Yabroff K.R., Lee D.J., Han X. (2021). Associations of Medicaid Expansion with Insurance Coverage, Stage at Diagnosis, and Treatment Among Patients with Genitourinary Malignant Neoplasms. JAMA Netw. Open.

[51] Jiang C., Perimbeti S., Deng L., Xing J., Chatta G.S., Han X., Gopalakrishnan D. (2023). Medicaid expansion and racial disparity in timely multidisciplinary treatment in muscle invasive bladder cancer. J. Natl. Cancer Inst..

[52] Ayanian J.Z., Weissman J.S. (2002). Teaching hospitals and quality of care: A review of the literature. Milbank Q..

[53] Zwiep T., Ahn S.H., Brehaut J., Balaa F., McIsaac D.I., Rich S., Wallace T., Moloo H. (2021). Group practice impacts on patients, physicians and healthcare systems: A scoping review. BMJ Open.

[54] Christmas C., Durso S.C., Kravet S.J., Wright S.M. (2010). Advantages and challenges of working as a clinician in an academic department of medicine: Academic clinicians’ perspectives. J. Grad. Med. Educ..

[55] Birkmeyer J.D., Siewers A.E., Finlayson E.V., Stukel T.A., Lucas F.L., Batista I., Welch H.G., Wennberg D.E. (2002). Hospital volume and surgical mortality in the United States. N. Engl. J. Med..

[56] Birkmeyer J.D., Stukel T.A., Siewers A.E., Goodney P.P., Wennberg D.E., Lucas F.L. (2003). Surgeon volume and operative mortality in the United States. N. Engl. J. Med..

[57] Lin C.C., Bruinooge S.S., Kirkwood M.K., Olsen C., Jemal A., Bajorin D., Giordano S.H., Goldstein M., Guadagnolo B.A., Kosty M. (2015). Association Between Geographic Access to Cancer Care, Insurance, and Receipt of Chemotherapy: Geographic Distribution of Oncologists and Travel Distance. J. Clin. Oncol..

[58] Lin C.C., Bruinooge S.S., Kirkwood M.K., Hershman D.L., Jemal A., Guadagnolo B.A., Yu J.B., Hopkins S., Goldstein M., Bajorin D. (2016). Association Between Geographic Access to Cancer Care and Receipt of Radiation Therapy for Rectal Cancer. Int. J. Radiat. Oncol. Biol. Phys..

[59] Massarweh N.N., Chiang Y.J., Xing Y., Chang G.J., Haynes A.B., You Y.N., Feig B.W., Cormier J.N. (2014). Association between travel distance and metastatic disease at diagnosis among patients with colon cancer. J. Clin. Oncol..

[60] Scoggins J.F., Fedorenko C.R., Donahue S.M., Buchwald D., Blough D.K., Ramsey S.D. (2012). Is distance to provider a barrier to care for medicaid patients with breast, colorectal, or lung cancer?. J. Rural Health.

[61] Ryan S., Serrell E.C., Karabon P., Mills G., Hansen M., Hayn M., Menon M., Trinh Q.D., Abdollah F., Sammon J.D. (2018). The Association between Mortality and Distance to Treatment Facility in Patients with Muscle Invasive Bladder Cancer. J. Urol..

[62] Lindberg M.H., Chen G., Olsen J.A., Abelsen B. (2022). Combining education and income into a socioeconomic position score for use in studies of health inequalities. BMC Public Health.

[63] Hasan S., Lazarev S., Garg M., Mehta K., Press R.H., Chhabra A., Choi J.I., Simone C.B., Gorovets D. (2023). Racial inequity and other social disparities in the diagnosis and management of bladder cancer. Cancer Med..

[64] Densmore R., Hajizadeh M., Hu M. (2019). Trends in socio-economic inequalities in bladder cancer incidence in Canada: 1992-2010. Can. J. Public Health.

[65] Christiansen K., Buswell L., Fadelu T. (2023). A Systematic Review of Patient Education Strategies for Oncology Patients in Low- and Middle-Income Countries. Oncologist.

